# Silencing circSLAMF6 represses cell glycolysis, migration, and invasion by regulating the miR-204-5p/MYH9 axis in gastric cancer under hypoxia

**DOI:** 10.1042/BSR20201275

**Published:** 2020-06-23

**Authors:** Xinhui Fang, Yangqiu Bai, Lida Zhang, Songze Ding

**Affiliations:** Department of Gastroenterology and Hepatology, Henan Provincial People’s Hospital, People’s Hospital of Zhengzhou University, School of Clinical Medicine, Henan University, Zhengzhou, Henan, 450003, China

**Keywords:** circSLAMF6, Gastric cancer, miR-204-5p, MYH9

## Abstract

**Background:** Gastric cancer (GC) is a malignant tumor of the digestive tract. Hypoxia plays an important role in the development of cancer, including GC. The present study aimed to investigate the role of circular RNA SLAMF6 (circSLAMF6) in the progression of GC under hypoxia.

**Methods:** The expression of circSLAMF6, microRNA-204-5p (miR-204-5p) and myosin heavy chain 9 (MYH9) was measured by quantitative real-time polymerase chain reaction (qRT-PCR). GC cells were maintained under hypoxia (1% O_2_) for experiments *in vitro*. Glucose consumption and lactate production were determined by a Glucose Assay Kit and a Lactate Assay Kit, respectively. Levels of all protein were detected by Western blot. Cell migration and invasion were examined by Transwell assay. The interaction between miR-204-5p and circSLAMF6 or MYH9 was analyzed by dual-luciferase reporter and RNA immunoprecipitation (RIP) assays. Murine xenograft model was established to explore the role of circSLAMF6 *in vivo*.

**Results:** CircSLAMF6 expression was increased in GC cells under hypoxia. Hypoxia promoted glycolysis, migration, and invasion in GC cells, which were reversed by circSLAMF6 knockdown. CircSLAMF6 was validated as a miR-204-5p sponge, and MYH9 was a target of miR-204-5p. Functionally, miR-204-5p inhibitor weakened the inhibition of circSLAMF6 knockdown on GC cell progression under hypoxia. Besides, MYH9 depletion suppressed glycolysis, migration, and invasion in GC cells under hypoxia. Importantly, circSLAMF6 deficiency inhibited tumor growth *in vivo* by regulating the miR-204-5p/MYH9 axis.

**Conclusion:** CircSLAMF6 was involved in glycolysis, migration, and invasion by regulating the miR-204-5p/MYH9 axis in GC cells under hypoxia.

## Introduction

Gastric cancer (GC) is a common malignancy, with more than 750000 additional cases annually worldwide [1]. In addition, the 5-year survival rate in advanced GC patients is also worrisome [2]. The pathogenesis of GC is complex and multifactorial. Hypoxia has been shown to play an important role in the development of solid tumors, which accelerates the spread of tumors, malignant progression, and increases the resistance to chemotherapy [3,4]. Under the condition of hypoxia, glycolysis can provide energy for cell metabolism and facilitate cell migration and invasion [5]. Therefore, it is necessary to study the mechanism of GC progression under hypoxia and explore novel biomarkers to assist early diagnosis of GC patients.

Circular RNAs (circRNAs) are a special type of non-coding RNAs (ncRNAs) with covalently closed loop structures, which were validated to be involved in many biological processes and the development of human cancers [6]. Additionally, circRNAs can participate in hypoxia-induced tumor progression. Su et al. revealed that circDENND2A could be induced by hypoxia in glioma cells, and it could mediate hypoxia-induced glioma cell migration and invasion through targeting miR-625-5p [7]. CircDENND4C inhibition impaired breast cancer cell glycolysis and metastasis via targeting miR-200b/c under hypoxia [8]. Furthermore, circRNA_403658 could potentiate the growth and glycolysis of bladder cancer cells under hypoxia by activating LDHA [9]. Circular RNA SLAMF6 (circSLAMF6, known as hsa_circ_0000144) is generated from the back splicing of SLAMF6 first intron. It has been indicated to play a carcinogenic role in bladder cancer by sponging miR-217 and regulating RUNX2 [10]. Importantly, a study demonstrated that circSLAMF6 could elevate the progression of GC cells [11]. However, the function and mechanism of circSLAMF6 in GC progression under hypoxic conditions remain unknown.

CircRNAs can perform their functions via serving as competitive endogenous RNA (ceRNA) for microRNAs (miRNAs) in different species [12]. Recent studies revealed that miR-204-5p played a tumor-inhibition role in laryngeal squamous cell carcinoma [13], non-small cell lung cancer [14], and melanoma [15]. MiR-204-5p also retarded the proliferation of GC cells by targeting USP47 and RAB22A [16], and Chen et al. reported that lncRNA LINC01234 facilitated GC cell growth by sponging miR-204-5p [17]. Myosin heavy chain 9 (MYH9) was implicated in a variety of biological processes, such as cell migration, differentiation, and morphogenesis [18]. In addition, high expression of MYH9 has been found to play an oncogenic role in ovarian cancer [19], esophageal squamous cancer [20], and GC [21]. However, the interaction among circSLAMF6, miR-204-5p and MYH9 in GC has not been reported till now.

In the present paper, we appraised circSLAMF6 expression in GC tissues and cells, and explored its effects on glycolysis, migration and invasion in GC cells under hypoxia. Meanwhile, we investigated the molecular mechanism of circSLAMF6 in GC cells under hypoxia.

## Materials and methods

### Tissue samples

Forty-five pairs of tumor tissues and the adjacent normal tissues were obtained from GC patients at Henan Provincial People’s Hospital, People’s Hospital of Zhengzhou University, School of Clinical Medicine, Henan University. None of the participants received any treatment before surgery. The collected samples were stored in liquid nitrogen. The research was carried out in accordance with the World Medical Association Declaration of Helsinki. All participants signed the written informed consents. The present study was approved by the ethics committee of the Henan Provincial People’s Hospital, People’s Hospital of Zhengzhou University, School of Clinical Medicine, Henan University.

### Cell culture and hypoxia stimulation

Human GC cell lines (AGS, MKN-45) and healthy gastric epithelial GES-1 cell line were acquired from Shanghai Institutes for Biological Sciences (Shanghai, China). Roswell Park Memorial Institute 1640 (RPMI 1640, Hyclone, South Logan, UT, U.S.A.) including 10% fetal bovine serum (FBS, Invitrogen, Carlsbad, CA, U.S.A.) was employed to hatch the cells at 37°C with 5% CO_2_. For hypoxia stimulation, AGS and MKN-45 cells were maintained in a hypoxia chamber with 1% O_2_ for various different times (0, 3, 6, 12, 24, and 48 h).

### Cell transfection

Small interfering RNA targeting circSLAMF6 (si-circSLAMF6) or MYH9 (si-MYH9) and the negative control (si-NC), miR-204-5p mimic (miR-204-5p) and its control (miR-NC), miR-204-5p inhibitor (anti-miR-204-5p) and its control (anti-miR-NC) were synthesized by RiboBio (Guangzhou, China). The lentiviral vector with interfering RNA sequence against circSLAMF6 (sh-circSLAMF6) and its control (sh-NC) was constructed by Wuyuan Company (Beijing, China). Cells were transfected with above constructed plasmids or oligonucleotides via Lipofectamine 3000 (Invitrogen).

### Quantitative real-time polymerase chain reaction

The RNA in GC cells and tissues was isolated by TRIzol solution (Invitrogen) and then synthesized into the complementary DNA (cDNA) by a PrimeScript RT Reagent Kit (Takara, Kusatsu, Shiga, Japan). The SYBR Premix Ex Taq™ (Takara) was used to carry out the quantitative real-time polymerase chain reaction (qRT-PCR). The expression of circSLAMF6 or MYH9 and miR-204-5p was normalized to glyceraldehyde-3-phosphate dehydrogenase (GAPDH) and U6, respectively. The $2^{-\text{ΔΔ}C_{\text{t}}}$ method was utilized to calculate the relative RNA expression. Primer sequences: circSLAMF6, forward, 5′-GAGTGTTGGCCTGTCCTCAA-3′, reverse, 5′-TTGTGCCCAGTTGCCTGTAT-3′; miR-204-5p, forward, 5′-CGAAGTTCCCTTTGTCATCCT-3′, reverse, 5′-GTGCAGGGTCCGAGGTATTC-3′; MYH9, forward, 5′-AGTTTGTCTCGGAGCTGTGG-3′, reverse, 5′-GGTTCGTGTTCCTCAGCGTA-3′; U6, forward, 5′-CGCTTCGGCAGCACATATAC-3′, reverse, 5′-AACGCTTCACGAATTTGCGT-3′; GAPDH, forward, 5′-GTGCACCTTGGTCCATTTG-3′, reverse, 5′-TGGTGAAGACGCCAGTGGA-3′.

### Glucose consumption and lactate production

The transfected AGS and MKN-45 cells were seeded into the six-well plates. Following the day, cells were cultured under hypoxia or normoxia for another 48 h. The cell medium was collected, and the concentrations of glucose and lactate were checked by using a Glucose Assay Kit and a Lactate Assay Kit (Sigma-Aldrich, St. Louis, MO, U.S.A.), respectively.

### Western blot

The protein in transfected GC cells or GC tissues was extracted by RIPA (Beyotime, Beijing, China). After high temperature denaturation at 98°C, sodium dodecyl sulfate polyacrylamide gel electrophoresis (SDS-PAGE) was utilized to separate the proteins, and the samples were then transferred to polyvinylidene fluoride (PVDF, Beyotime) membranes. The membrane was mixed with 5% skim milk for 2 h and treated with the primary antibodies against hexokinase 2 (HK2, 1:2000, Abcam, Cambridge, MA, U.S.A.), matrix metallopeptidase 2 (MMP2, 1:1000, Abcam), MMP9 (1:1000, Abcam), MYH9 (1:1000, Cell Signaling Technology, Danvers, MA, U.S.A.), or GAPDH (1:2000, Abcam) at 4°C overnight. Horseradish peroxidase-conjugated the antibodies anti-rabbit immunoglobulin G (IgG) (1:5000, Cell Signaling Technology) were used to incubate the membranes. The protein bands were visualized by using a BeyoECL Plus Kit (Beyotime).

### Transwell assay

The migration and invasion of AGS and MKN-45 cells were evaluated through Transwell assay with the chamber. Differently, the upper chamber was needed to coat with Matrigel (BD Bioscience, San Jose, CA, U.S.A.) when cell invasion detection was performed. The transfected cells cultured in serum free medium were added into the upper chamber in the 24-Transwell plates, and 600 μl medium containing 10% FBS was added into the lower chamber. After 24 h transfection, the migrated or invaded cells were stained with 0.1% crystal violet. Twenty minutes later, an inverted microscope was utilized to photograph and count the cells.

### Dual-luciferase reporter assay

To affirm circSLAMF6 could bind to miR-204-5p and miR-204-5p directly targeted MYH9, circSLAMF6 or MYH9 wild-type reporter vector (circSLAMF6-WT or MYH9 3′UTR-WT) containing miR-204-5p binding sites and their mutated-type reporter vectors (circSLAMF6-MUT or MYH9 3′UTR-MUT) without binding sites were constructed. These reporter vectors were co-transfected into AGS and MKN-45 cells with miR-204-5p or miR-NC by Lipofectamine 3000. After 24 h post-transfection, the luciferase activity was estimated through a Dual-luciferase Reporter Assay System (Promega, Madison, WI, U.S.A.).

### RNA immunoprecipitation assay

An EZ-Magna RIP Kit (Millipore, Billerica, MA, U.S.A.) was employed to confirm the interaction between miR-204-5p and circSLAMF6 or MYH9 in RNA immunoprecipitation (RIP) assay. Briefly, AGS and MKN-45 cells were transfected with miR-204-5p or miR-NC and then cultured for 48 h. Cells were harvested and lysed in RIP lysis buffer. The magnetic beads pre-coated with Argonaute-2 (Ago2) antibody or IgG antibody were incubated with the cells overnight at 4°C. The RNA on the magnetic beads was purified and extracted, and the level of circSLAMF6 or MYH9 enriched by RIP was examined by qRT-PCR.

### Tumor xenograft mice

To establish xenograft tumor model, AGS cells transfected with sh-circSLAMF6 or sh-NC were injected into the male nude mice (4–5 weeks old, *n* = 5 per group). Tumor volume was monitored once a week. Five weeks after injection, the nude mice were all killed and the tumors were collected and weighed. Abundance of circSLAMF6, miR-204-5p, or MYH9 in tumor samples was determined by qRT-PCR or Western blot. The experiments were carried out in the Henan Provincial People’s Hospital, People’s Hospital of Zhengzhou University, School of Clinical Medicine, Henan University. Mice were killed by cervical dislocation after deep anesthesia with 2% isoflurane. Animal studies were performed in compliance with the ARRIVE guidelines and the Basel Declaration. All animals received humane care according to the National Institutes of Health (U.S.A.) guidelines. The experiment was permitted by the Animal Care and Use Committee of Henan Provincial People’s Hospital, People’s Hospital of Zhengzhou University, School of Clinical Medicine, Henan University.

### Statistical analysis

Data were shown as mean ± standard deviation (SD) and analyzed using Graph-pad prism 7.0 tool. Each experiment was repeated at least three times. The comparison between two or more groups was evaluated by using Chi-square test or one-way analysis of variance (ANOVA). Kaplan–Meier survival assay and log-rank test were used to assess the relationship between circSLAMF6 level and prognosis of GC patients. The correlation among circSLAMF6, miR-204-5p, and MYH9 in GC tissues was analyzed by Pearson correlation analysis. *P*<0.05 was regarded as significant difference.

## Results

### The abundance of circSLAMF6 was up-regulated in GC cells under hypoxia

**Figure 1:**
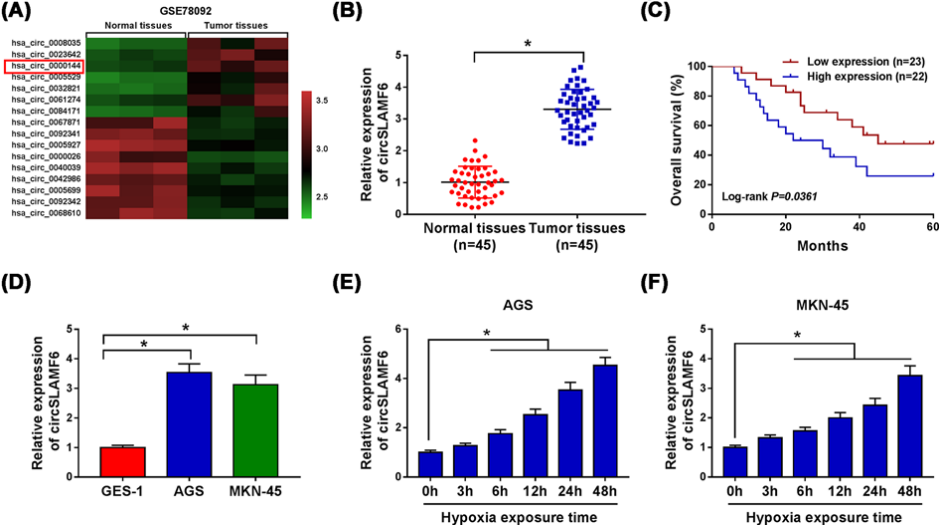
The abundance of circSLAMF6 was up-regulated in GC cells under hypoxia (**A**) The heatmap showed that the differentially expressed circRNAs in GC tissues and paired adjacent normal tissues. (**B**) CircSLAMF6 expression in 45 GC tissues and the adjacent normal tissues was measured by qRT-PCR. (**C**) Kaplan–Meier survival curves and log-rank tests were used to explore the relationship between circSLAMF6 expression and overall survival of GC patients. (**D**) CircSLAMF6 expression in GC cell lines (AGS and MKN-45) and normal cells (GES-1) was examined by qRT-PCR. (**E,F**) Relative expression of circSLAMF6 in AGS and MKN-45 cells after hypoxia exposure for various times was detected by qRT-PCR. *P<0.05.

To identify GC-related circRNAs, we analyzed the GEO database (GSE78092) using the GEO2R method. The top seven up-regulated and nine down-regulated circRNAs in GC tissue were presented in Figure 1A by the hierarchical clustering. Through literature search, we chose hsa_circ_0000144 (circSLAMF6) for further investigation. To explore the role of circSLAMF6 in GC, its expression was examined in GC tissues. As shown in Figure 1B, qRT-PCR data showed that circSLAMF6 expression was drastically increased in tumor tissues compared with the corresponding adjacent normal tissues. Kaplan–Meier survival curves showed that the patients in high circSLAMF6 level group had a shorter survival time than those in low circSLAMF6 level group (Figure 1C). Importantly, circSLAMF6 expression was significantly increased in the two CC cell lines (AGS and MKN-45) that in healthy gastric epithelial GES-1 cell line (Figure 1D). Furthermore, circSLAMF6 expression in AGS and MKN-45 cells after exposure of hypoxia was measured. The results indicated that the expression of circSLAMF6 was progressively induced in AGS and MKN-45 cells after exposure of hypoxia in a time dependent manner (Figure 1E,F). These results demonstrated that circSLAMF6 might implicate in GC progression.

### Silencing circSLAMF6 impeded glycolysis of GC cells under hypoxia

**Figure 2:**
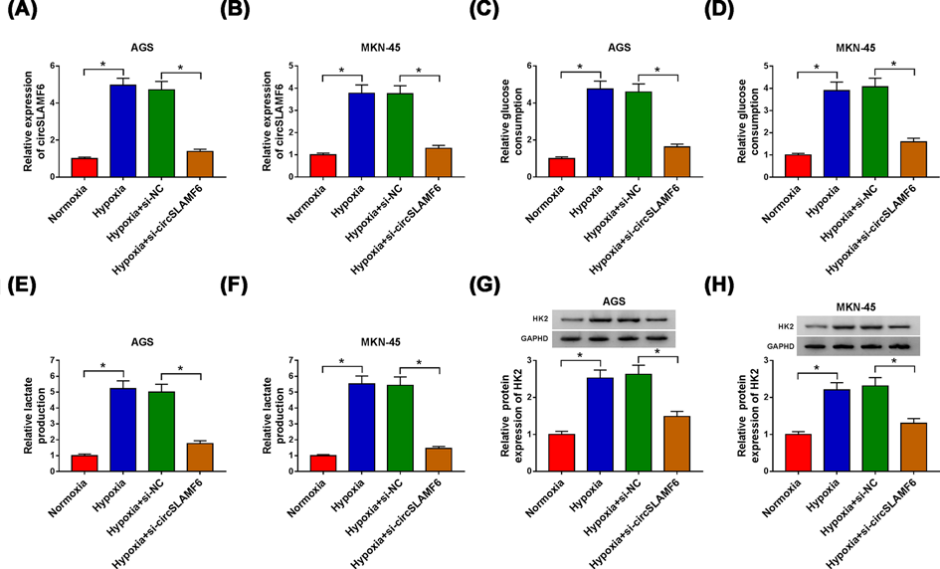
Silencing circSLAMF6 impeded glycolysis of GC cells under hypoxia AGS and MKN-45 cells were transfected with si-NC or si-circSLAMF6 after treatment of hypoxia for 48 h. (**A,B**) CircSLAMF6 expression was measured by qRT-PCR. (**C-F**) Glucose consumption and lactate production were determined by the Glucose Assay Kit and Lactate Assay Kit, respectively. (**G,H**) HK2 protein expression was detected via Western blot. *P<0.05.

Based on the results mentioned above, the effect of circSLAMF6 knockdown on glycolysis of GC cells under hypoxia was explored. As shown in Figure 2A,B, circSLAMF6 level was augmented in AGS and MKN-45 cells under hypoxia of 48 h, and transfection of si-circSLAMF6 reduced the level of circSLAMF6. Meanwhile, we found that glucose consumption (Figure 2C,D), lactate production (Figure 2E,F), and HK2 protein expression (Figure 2G,H) in hypoxia-induced AGS and MKN-45 cells were obviously induced, however, knockdown of circSLAMF6 enormously attenuated these effects, suggesting that circSLAMF6 inhibition could hinder glycolysis of GC cells under hypoxia.

### CircSLAMF6 depletion inhibited cell migration and invasion in GC cells under hypoxia

**Figure 3:**
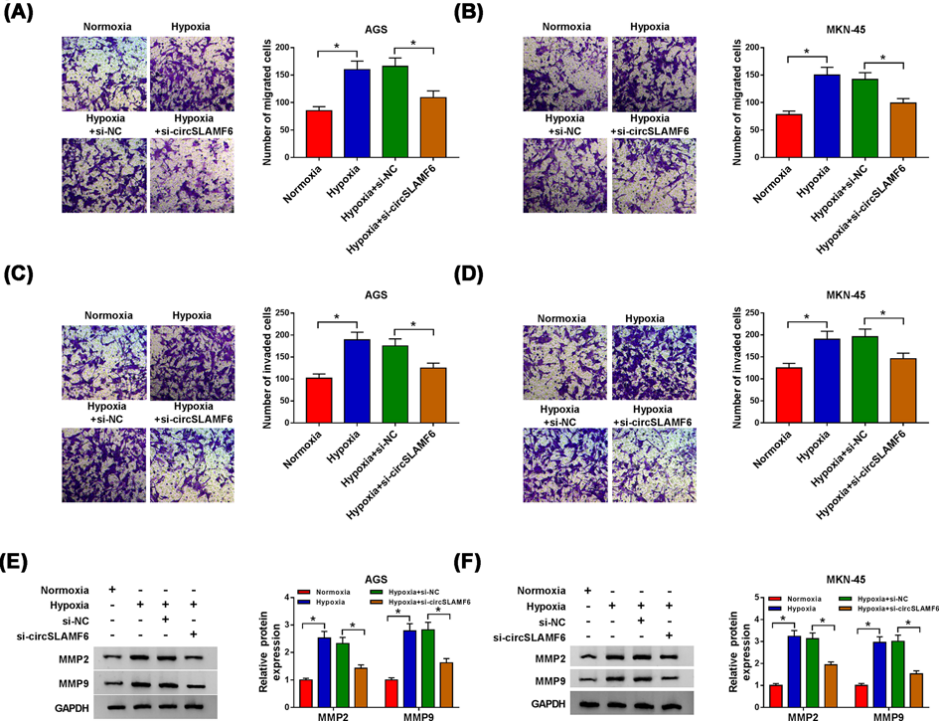
CircSLAMF6 depletion inhibited cell migration and invasion in GC cells under hypoxia AGS and MKN-45 cells were transfected with si-NC or si-circSLAMF6 after treatment of hypoxia for 48 h. (**A–D**) Cell migration and invasion were examined by Transwell assay. (**E,F**) The protein expression levels of MMP2 and MMP9 were measured by Western blot. *P<0.05.

We then investigated the effects of circSLAMF6 on GC cell migration and invasion, and Transwell assay was performed. The findings manifested that the promotion effects of hypoxia on cell migration and invasion were weakened by interfering with circSLAMF6 in AGS and MKN-45 cells (Figure 3A–D). Expression levels of key proteins MMP2 and MMP9 associated with migration and invasion were also detected by Western blot. MMP2 and MMP9 expression levels in AGS and MKN-45 cells were boosted by hypoxia exposure, while these effects were neutralized by silencing circSLAMF6 (Figure 3E,F). The results supported that circSLAMF6 knockdown suppressed migration and invasion of GC cells under hypoxia.

### CircSLAMF6 acted as a sponge of miR-204-5p in GC cells

**Figure 4:**
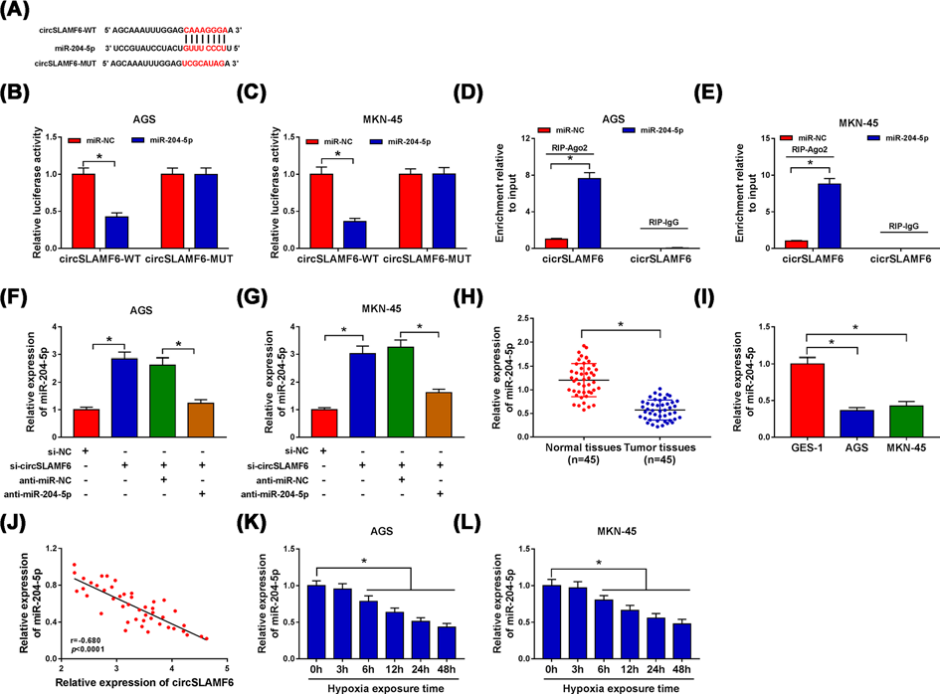
CircSLAMF6 acted as a sponge of miR-204-5p in GC cells (**A**) The binding sites between circSLAMF6 and miR-204-5p were predicted by circBank. (**B–E**) Dual-luciferase reporter assay and RIP assay were performed to verify the interaction between circSLAMF6 and miR-204-5p in AGS and MKN-45 cells. (**F,G**) Relative expression of miR-204-5p in AGS and MKN-45 cells transfected with si-NC, si-circSLAMF6, si-circSLAMF6 + anti-miR-NC, or si-circSLAMF6 + anti-miR-204-5p was examined by qRT-PCR. (**H,I**) Relative expression of miR-204-5p in GC tissues and cells was determined by qRT-PCR. (**J**) The correlation between circSLAMF6 and miR-204-5p expression in GC tissues was analyzed by Pearson correlation analysis. (**K,L**) The abundance of miR-204-5p in AGS and MKN-45 cells after hypoxia exposure for various times was detected by qRT-PCR. *P<0.05.

Numerous studies indicated that circRNAs can function as sponges for miRNA to exert their biological effects [22]. To search for circSLAMF6-associated miRNAs, circBank bioinformatics analysis was employed. As shown in Figure 4A, there were complementary binding sites between circSLAMF6 and miR-204-5p. Dual-luciferase reporter assay and RIP assay were then performed. The results showed that miR-204-5p overexpression in AGS and MKN-45 cells obviously decreased the luciferase activity of circSLAMF6-WT, but had no significant effect on the luciferase activity of circSLAMF6-MUT (Figure 4B,C). RIP assay demonstrated that transfection of miR-204-5p resulted in the enrichment of circSLAMF6 in RIP-Ago2 group, however, there was almost no enrichment of circSLAMF6 in IgG group (Figure 4D,E). In addition, knockdown of circSLAMF6 elevated the expression of miR-204-5p in AGS and MKN-45 cells, and the effect was abolished by co-transfection with anti-miR-204-5p (Figure 4F,G). The results showed that miR-204-5p expression was prominently decreased in GC tissues and cells (AGS and MKN-45) compared with normal tissues and cells (GES-1) (Figure 4H,I). A negative correlation between circSLAMF6 and miR-204-5p expression in GC tissues was also observed (Figure 4J). Moreover, miR-204-5p expression in hypoxia-induced AGS and MKN-45 cells was progressively degraded in a time dependent manner (Figure 4K,L). These data revealed that the expression of miR-204-5p was down-regulated in GC cells under hypoxia and inversely modulated by circSLAMF6.

### Silencing circSLAMF6 impaired glycolysis, migration, and invasion in GC cells under hypoxia by targeting miR-204-5p

**Figure 5:**
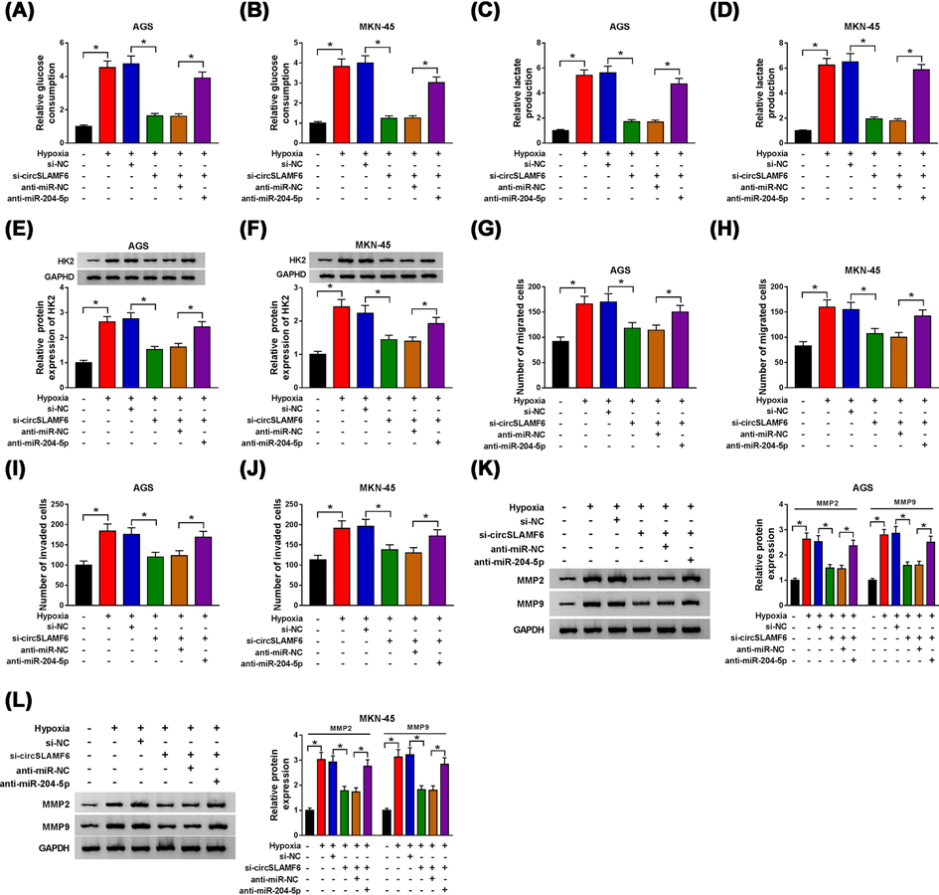
Silencing circSLAMF6 impaired glycolysis, migration, and invasion in GC cells under hypoxia by targeting miR-204-5p AGS and MKN-45 cells were transfected with si-NC, si-circSLAMF6, si-circSLAMF6 + anti-miR-NC, or si-circSLAMF6 + anti-miR-204-5p after treatment of hypoxia. (**A–D**) Glucose consumption and lactate production were assessed by using the Glucose Assay Kit and Lactate Assay Kit, respectively. (**E,F**) The protein expression of HK2 was measured by Western blot. (**G–J**) Transwell assay was carried out to analyze cell migration and invasion. (**K,L**) The protein levels of MMP2 and MMP9 were examined via Western blot. *P<0.05.

To explore whether miR-204-5p affected the role of circSLAMF6 in GC, AGS, and MKN-45 cells were co-transfected with si-circSLAMF6 and anti-miR-204-5p under hypoxia. The results manifested that the inhibitory effects of circSLAMF6 suppression on glucose consumption (Figure 5A,B), lactate production (Figure 5C,D) and HK2 protein expression (Figure 5E,F) could be rescued by interfering with miR-204-5p in AGS and MKN-45 cells under hypoxia. Simultaneously, miR-204-5p deficiency could reverse the inhibition of si-circSLAMF6 on migration (Figure 5G,H) and invasion (Figure 5I,J) in AGS and MKN-45 cells under hypoxia. Moreover, the levels of MMP2 and MMP9 reduced by circSLAMF6 knockdown were ameliorated via miR-204-5p repression (Figure 5K,L). These results revealed that circSLAMF6 knockdown inhibited GC cell progression under hypoxia by increasing miR-204-5p.

### CircSLAMF6 could regulate the expression of MYH9 targeted by miR-204-5p

**Figure 6:**
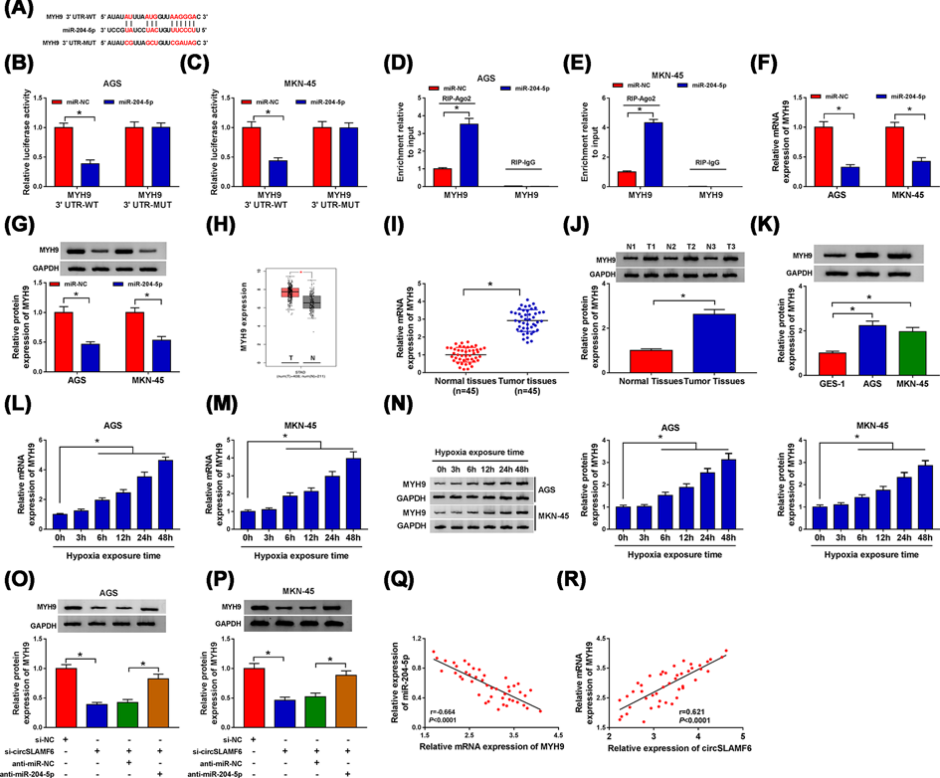
CircSLAMF6 could regulate the expression of MYH9 targeted by miR-204-5p (**A**) The binding sites between miR-204-5p and MYH9 were predicted using StarBase 3.0 database. (**B–E**) Dual-luciferase reporter assay and RIP assay were utilized to confirm the interaction between miR-204-5p and MYH9 in AGS and MKN-45 cells. (**F,G**) Both mRNA and protein levels of MYH9 in AGS and MKN-45 cells transfected with miR-NC or miR-204-5p were determined by qRT-PCR and Western blot, respectively. (**H**) MYH9 mRNA expression in GC tumor tissues and normal tissues was analyzed download from TCGA database. (**I,J**) The mRNA and protein levels of MYH9 in GC tissues and cells were assessed by qRT-PCR and Western blot, respectively. (**K**) The protein expression of MYH9 in GC cells (AGS and MKN-45) and normal cells (GES-1) was measured by Western blot. (**L-N**) The mRNA and protein levels of MYH9 in AGS and MKN-45 cells after hypoxia exposure for various times were evaluated by qRT-PCR and Western blot, respectively. (**O,P**) The protein expression of MYH9 in AGS and MKN-45 cells transfected with si-NC, si-circSLAMF6, si-circSLAMF6 + anti-miR-NC, or si-circSLAMF6 + anti-miR-204-5p was examined by Western blot. (**Q,R**) The correlation between MYH9 and miR-204-5p or circSLAMF6 expression in GC tissues was analyzed using Pearson correlation analysis. *P<0.05.

Subsequently, we used StarBase 3.0 database to search for the downstream targets of miR-204-5p. The results showed that there were miR-204-5p binding sites in the 3′UTR of MYH9 (Figure 6A). Moreover, overexpression of miR-204-5p distinctly degraded the luciferase activity of MYH9 3′UTR-WT, but had no significant effect on the luciferase activity of MYH9 3′UTR-MUT in AGS and MKN-45 cells (Figure 6B,C). As expected, the enrichment of MYH9 in miR-204-5p transfected AGS and MKN-45 cells by RIP-Ago2 was higher, whereas the RIP-IgG group showed little enrichment of MYH9 (Figure 6D,E). The results suggested that MYH9 was the target of miR-204-5p. Besides, miR-204-5p overexpression drastically reduced both mRNA and protein expression of MYH9 in AGS and MKN-45 cells (Figure 6F,G). As presented in Figure 6H, the abundance of MYH9 was found to be up-regulated in GC tumor tissues compared with normal tissues from TCGA database. Consistently, the mRNA and protein levels of MYH9 in GC tissues were significantly increased than that in normal tissues (Figure 6I,J), and the protein expression of MYH9 in GC cell lines was also boosted (Figure 6K). Furthermore, both mRNA and protein expression levels of MYH9 were progressively elevated in AGS and MKN-45 cells after hypoxia stimulation in a time dependent manner (Figure 6L–N). We also found that knockdown of circSLAMF6 could decline the protein expression of MYH9 in the two cells, while miR-204-5p inhibition reversed this effect (Figure 6O,P). Importantly, MYH9 expression strongly negatively correlated with miR-204-5p expression, while positively correlated with circSLAMF6 expression in GC tissues (Figure 6Q,R). Taken together, these data suggested that circSLAMF6 might regulate MYH9 expression by acting as a sponge for miR-204-5p.

### Knockdown of MYH9 curbed glycolysis, migration, and invasion in GC cells under hypoxia

**Figure 7:**
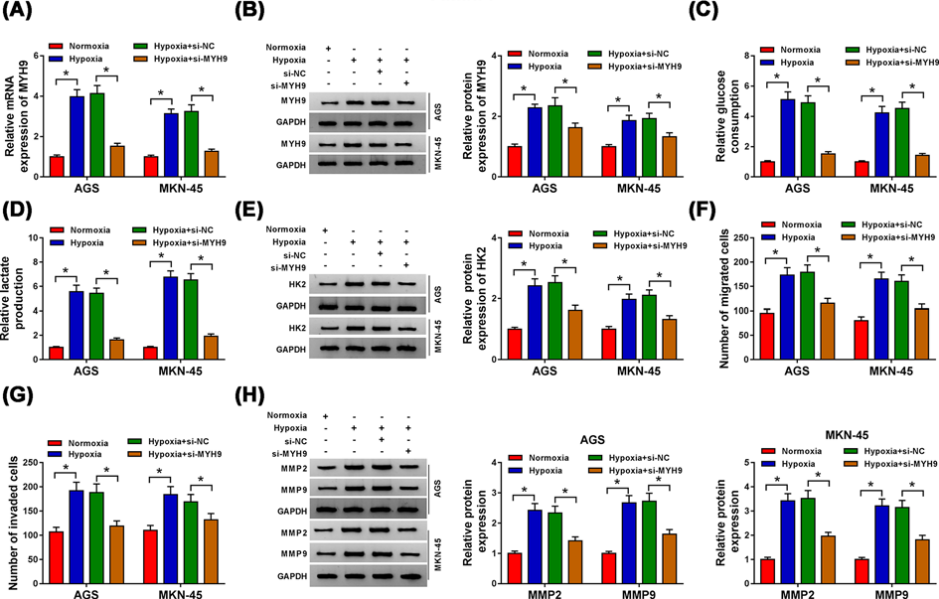
Knockdown of MYH9 curbed glycolysis, migration, and invasion in GC cells under hypoxia AGS and MKN-45 cells were transfected with si-NC or si-MYH9 after treatment of hypoxia. (**A,B**) The mRNA and protein levels of MYH9 were determined by qRT-PCR and Western blot, respectively. (**C–E**) Glucose consumption, lactate production, and HK2 protein expression were estimated by the Glucose Assay Kit, Lactate Assay Kit and Western blot, respectively. (**F,G**) Cell migration and invasion were evaluated by Transwell assay. (**H**) The protein expression levels of MMP2 and MMP9 were checked by Western blot. *P<0.05.

To investigate the possible effects of MYH9 on cell glycolysis, migration, and invasion under hypoxia, si-MYH9 or si-NC was transfected into AGS and MKN-45 cells under hypoxia. As shown in Figure 7A,B, both mRNA and protein expression of MYH9 in AGS and MKN-45 cells were significantly up-regulated after hypoxia stimulation, which was reversed by transfection of si-MYH9. Then, we observed that MYH9 knockdown markedly alleviated the effects on glucose consumption (Figure 7C), lactate production (Figure 7D) and HK2 protein expression (Figure 7E) in AGS and MKN-45 cells under hypoxia. Transwell assay data further showed that si-MYH9 transfection strikingly suppressed cell migration and invasion ability of AGS and MKN-45 cells facilitated by hypoxia (Figure 7F,G). Moreover, the promotion effects of hypoxia on the protein levels of MMP2 and MMP9 were abolished by MYH9 depletion (Figure 7H). These results supported that MYH9 inhibition impeded glycolysis, migration and invasion in GC cells under hypoxia.

### Silencing circSLAMF6 inhibited tumor growth *in vivo*

**Figure 8:**
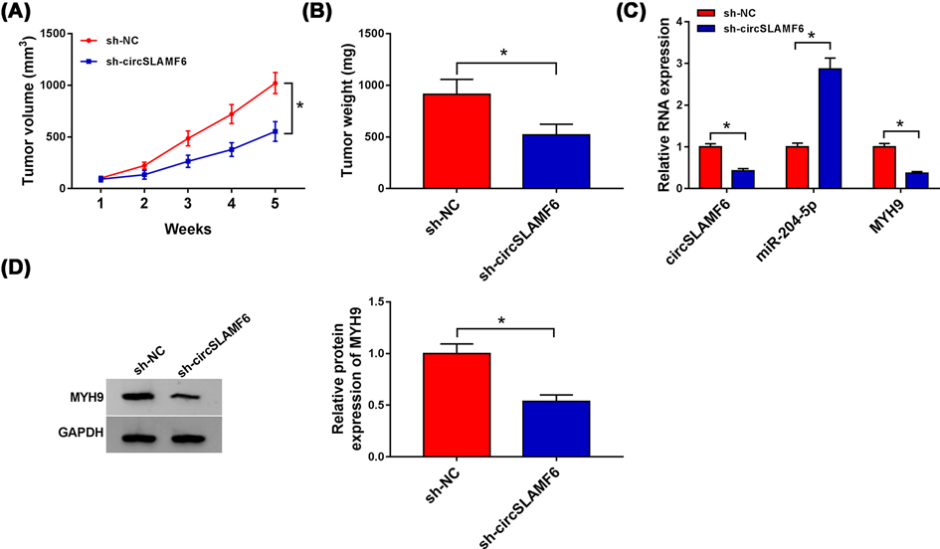
Silencing circSLAMF6 inhibited tumor growth *in vivo* AGS cells stably transfected with sh-circSLAMF6 or sh-NC were subcutaneously implanted into nude mice (*n* = 4 each group). Five weeks later, all mice were killed. (**A,B**) Tumor volume and weight were measured. (**C**) The levels of circSLAMF6, miR-204-5p, and MYH9 in xenograft tissues were detected by qRT-PCR. (**D**) MYH9 protein expression in xenograft tissues was examined by Western blot. *P<0.05.

Next, mice xenograft models of GC were established to explore the role of circSLAMF6 *in vivo*. The results revealed that tumor volume and weight were obviously reduced in sh-circSLAMF6 group compared with those in sh-NC group (Figure 8A,B). Meanwhile, circSLAMF6 and MYH9 (mRNA and protein levels) were distinctly reduced, while miR-204-5p expression was highly expressed in tumor tissues derived from sh-circSLAMF6 group (Figure 8C,D). These findings validated that circSLAMF6 knockdown could hinder tumor growth by regulating the miR-204-5p/MYH9 axis.

## Discussion

GC ranks the third most common causes of cancer-related deaths globally [23]. Hypoxia is an essential feature in the neoplastic microenvironment. CircRNA was found to participate in the modulation of tumor progression under hypoxia [24], but there were few studies on circRNAs that affected the occurrence and development of GC under hypoxia.

CircRNAs are a special class of RNA molecules, which are highly stable and conserved among species due to their closed circular structures [25]. It has been shown that several circRNAs, such as circRACGAP1 [26] and circHIPK3 [27], can regulate the development of GC. In the present study, through GEO database analysis, circSLAMF6 showed higher expression in GC tissues compared with normal tissues. Our results further indicated that circSLAMF6 was significantly elevated in GC tissues and cell lines, and these results were consistent with those of Wei et al. [11]. Moreover, we observed that circSLAMF6 expression was augmented in GC cells under hypoxia. Importantly, knockdown of circSLAMF6 could restrain cell glycolysis, migration and invasion in GC cells under hypoxia. These results revealed that circSLAMF6 played an important role in promoting the development of malignant behaviors in GC, suggesting that circSLAMF6 might be a potential molecular target for GC therapy.

CircRNA can regulate mRNA expression by serving as a miRNA sponge [28]. To appraise whether circSLAMF6 modulate GC progression via sponging miRNAs, circBank bioinformatics analysis was used, and miR-204-5p was validated to be the target of circSLAMF6 by dual-luciferase activity and RIP assays. Many researchers revealed that miR-204-5p implicated in the progression of GC. Liang et al. reported that DLX6-AS1 boosted cell proliferation and metastasis by targeting miR-204-5p in GC [29]. Huan et al. found that miR-204-5p hindered GC cell proliferation, motility, and invasion [30]. Moreover, lncRNA KCNQ1OT1 could aggravate hypoxia-induced cardiac injury *in vitro* by targeting miR-204-5p [31]. In our results, miR-204-5p was remarkably reduced in GC cells under hypoxia. Besides, circSLAMF6 could negative modulate miR-204-5p expression, inversely, interference of miR-204-5p reversed the effects of circSLAMF6 knockdown on GC cell glycolysis, migration, and invasion under hypoxia. These data implied that circSLAMF6 played a carcinogenic role by inhibiting the expression of miR-204-5p in GC cells.

Then, MYH9 was identified as the downstream target of miR-204-5p. MYH9 is a gene encoding nonmuscle myosin IIA (NMIIA), belongs to the myosin II subfamily, and it plays an essential role in invasion and metastasis of tumor cells [32,33]. In this research, MYH9 expression was drastically boosted in GC cells under hypoxia. Furthermore, circSLAMF6 knockdown reduced MYH9 expression by combining with

miR-204-5p, and the promotion of hypoxia on cell progression could be neutralized by MYH9 suppression, suggesting that MYH9 could also play a oncogenic role in GC development, which was in agreement with the previous reports [34]. Additionally, *in vivo* experiments showed that circSLAMF6 depletion could inhibit tumor growth by decreasing MYH9 and increasing miR-204-5p.

In summary, our results revealed that circSLAMF6 knockdown played an anti-cancer role by inhibiting cell glycolysis, migration, and invasion through sponging miR-204-5p and regulating MYH9 in GC under hypoxia, providing a novel insight into the pivotal role of circRNA-miRNA-mRNA functional network in GC development.

## Abbreviations

Ago2: argonaute-2
ceRNA: competitive endogenous RNA
circSLAMF6: circular RNA SLAMF6
FBS: fetal bovine serum
GAPDH: glyceraldehyde-3-phosphate dehydrogenase
GC: gastric cancer
HK2: hexokinase 2
IgG: immunoglobulin G
MMP2: matrix metallopeptidase 2
MYH9: myosin heavy chain 9
ncRNA: non-coding RNA
qRT-PCR: quantitative real-time polymerase chain reactio
RIP: RNA immunoprecipitation
